# Supporting Web-Based Teaching and Learning of Virtual Care Skills and Competencies: Development of an Evidence-Informed Framework

**DOI:** 10.2196/75868

**Published:** 2025-07-03

**Authors:** Lorelli Nowell, Sara Dolan, Sonja Johnston, Michele Jacobsen, Diane L Lorenzetti, Elizabeth Oddone Paolucci

**Affiliations:** 1 Faculty of Nursing, University of Calgary, 2500 University Dr NW, Calgary, AB, T2N 1N4, Canada, 1 4036209822; 2 Werklund School of Education, University of Calgary, Calgary, AB, Canada; 3 Department of Community Health Sciences, Cumming School of Medicine, University of Calgary, Calgary, AB, Canada; 4 Health Sciences Library, University of Calgary, Calgary, AB, Canada; 5 Department of Surgery, Cumming School of Medicine, University of Calgary, Calgary, AB, Canada

**Keywords:** virtual care, telehealth, telemedicine, education, distance, clinical competence, empathy, patient-centered care, educational technology, curriculum, health education

## Abstract

**Background:**

Professionals across caring disciplines have played a significant role in the development of virtual care, which uses remote technologies to offer support and services from a distance. As virtual care becomes increasingly essential, instructors must ensure that students are equipped with both interpersonal abilities and digital competencies, merging traditional hands-on methods with web-based learning. Despite its growing importance, there is a lack of comprehensive frameworks to guide the design and delivery of web-based learning experiences that foster the development of virtual caring skills and competencies among students in caring professions.

**Objective:**

This study aims to develop an evidence-informed framework to support web-based teaching and learning of virtual caring skills and competencies.

**Methods:**

We present a synthesis of our research resulting in an evidence-informed framework. We integrated findings from an evidence synthesis, surveys, and semistructured interviews with students and educators, and consultations with key stakeholders.

**Results:**

Principles of this framework include (1) connection and interaction; (2) compassion, empathy, and care; (3) vulnerability; (4) a client-centered focus; (5) inclusivity and accessibility; and (6) flexibility. The framework’s four main domains are (1) virtual caring skills; (2) teaching and learning methods; (3) barriers to teaching, learning, and providing virtual care; and (4) facilitators of teaching, learning, and providing virtual care.

**Conclusions:**

This framework was developed by and for students and educators to aid in planning, promoting, and enhancing virtual caring skills development. It can be used to better equip students to provide virtual care, thereby positively impacting client care and outcomes. This framework can support educators, students, decision makers, and practice partners to build learning experiences aimed at preparing students to provide virtual care effectively.

## Introduction

### Background

Professionals across caring disciplines, including educators, doctors, nurses, social workers, and allied health professionals, have played a crucial role in the global response to COVID-19 pandemic. The rapid shift toward virtual care, where care is provided remotely through information technologies, has placed an unparalleled burden on these caring professionals [1,2]. With virtual care practices now essential for health care delivery, it is imperative to educate current students in caring professions and expose them to the requisite interpersonal and technological competencies for effective virtual care. However, there are no comprehensive frameworks to support the development and implementation of web-based learning opportunities that help students in caring professions develop the required virtual caring skills and competencies.

### Caring Professional Education

Caring professions, such as education, medicine, nursing, social work, and other allied health professions, are dedicated to supporting the health, well-being, development, education, and social needs of individuals and communities. These professions prioritize humanitarian objectives over material needs. Traditionally, education in caring professions relied on conventional methods such as face-to-face lectures, experiential in-class learning, and seminar formats. These educational approaches were often complemented by work-integrated learning placements, where students engaged with seasoned educators and practicing health professionals to acquire essential hands-on skills, dispositions, and competencies (eg, K-12 classrooms, hospital settings, and counseling centers) [3,4]. The landscape of education has evolved in caring professions due to advancements in technology and the emergence of virtual care. The integration of technology into education has opened new avenues for learning and skill development through web-based platforms, simulations, and virtual reality, for example, which supplement traditional teaching methods. The shifts to web-based and blended learning models have placed growing emphasis on fostering competencies related to virtual care, including effective communication through digital platforms, ethical considerations in telehealth, and leveraging technology to enhance client experiences and outcomes. While web-based education and some aspects of virtual care existed prior to the COVID-19 pandemic, the pandemic required everyone, irrespective of profession, location, or preference, to find new and inclusive ways of integrating web-based education and virtual care.

### Impact of COVID-19 Pandemic

The COVID-19 pandemic necessitated an abrupt shift and expansion to virtual teaching, learning, and caring, prompting caring professional degree programs to adopt alternative strategies for providing students with necessary training experiences and learning opportunities [1,5,6]. This sudden transition placed unprecedented demands on higher education institutions to effectively instruct core competencies crucial to care provision and on caring professionals to ensure the meaningful implementation of virtual care in practice [1,4]. Despite long-standing literature emphasizing the need to support educators in meeting students’ requirements [7,8], this need has become even more critical in the context of the COVID-19 pandemic [9].

This global crisis brought about significant changes in educational and care delivery methods, underscoring the importance of equipping caring professionals with the necessary skills to navigate current challenges and future uncertainties. Many educators in the caring professions encountered challenges in integrating effective web-based learning experiences, grappling with both technological and pedagogical hurdles as they prepared students for virtual work environments [10-13]. The pandemic prompted higher education institutions to reassess web-based education delivery within the caring professions and identify essential technological competencies for success in today’s digital economy. The onset of COVID-19 pandemic emphasized the necessity for a structured, evidence-based approach to develop and implement educational technologies in web-based teaching and learning contexts [2,10,14,15].

While some promising adaptations and strategies have been reported globally by higher education institutions [12,16-18], there is currently no empirical framework specifically designed for the web-based teaching and learning environment of caring professional education. Recent research underscores the need for such a framework to better understand the conditions fostering productive e-learning opportunities and to establish structured educational goals and methodologies that support the development of students’ virtual caring skills [11,19-21].

While there is a pressing need for educators to promote the development of both interpersonal and digital skills essential for providing virtual care, incorporating digital technologies into web-based classrooms and virtual caring professional practice can increase the expenses, time commitment, energy, and effort needed to provide students with substantial educational experiences. Despite practical and educational hurdles associated with emerging technologies, faculty members can overcome these barriers by adopting a deliberate and strategic approach to their work. To our knowledge, no comprehensive frameworks have been developed or evaluated to outline the necessary conditions to create effective practices to foster productive e-learning opportunities to support the development of virtual care skills and competencies for students in caring professions.

### Research Aims

The main aim of this research was to develop an evidence-informed framework supporting the teaching and learning of virtual caring skills and competencies. This framework can be used by (1) educators for planning, implementing, and evaluating teaching and learning strategies aimed at developing virtual caring skills and competencies; (2) students to assess their strengths and areas needing improvement in virtual caring skills and competencies; (3) institutional decision makers to inform key strategies related to teaching and learning virtual caring skills and competencies; and (4) employers to gain insight into the broad range of virtual caring skills and expertise that caring professionals can contribute to the workforce.

## Methods

### Data Sources

We used an integrated knowledge translation approach [22] to develop our framework using a collaborative research model where researchers and knowledge users, including educators and students, worked together. Our multidisciplinary team included 2 students and 4 faculty members from diverse caring professions within the same institution. Together, we codeveloped the research questions, successfully secured research funding, and actively participated throughout the framework development process. In using this approach, which involved shared power and coproduction of knowledge, we aimed to generate more relevant and actionable findings with greater uptake across policy and practice. Our research used a mixed methods pragmatic approach [23], incorporating (1) evidence synthesis, (2) surveys involving students and educators, (3) semistructured interviews with students and educators, and (4) consultations with key stakeholders.

### Evidence Synthesis

We conducted a systematic review to identify web-based learning opportunities aimed at helping students in caring professions to develop and apply virtual caring skills and competencies in virtual caring environments. Our synthesis encompassed evidence from 38 studies, curriculum details, study outcomes, barriers and facilitators to technology integration, impacts on students, and effects on professional practice [24]. The findings from our review guided the formulation of surveys for students and educators, followed by subsequent semistructured interviews.

### Surveys

Based on the results of our systematic review, we developed 2 surveys, 1 for students and 1 for educators, from caring professions within the same institution. These surveys included demographic items and questions about experiences with and perceptions of web-based teaching and learning technologies, instructional methods, and virtual caring skills. Items included closed and open text responses. Survey data were analyzed using descriptive statistics, with variations in distributions across the dataset summarized and presented in tables and graphs [25]. Inferential statistics were used to investigate potential relationships between demographic variables and satisfaction, preparedness, and the likelihood of using web-based teaching and learning technologies for developing virtual caring skills [25]. Open text responses were analyzed with interview data. There were 93 student and 82 educator survey respondents. The complete survey results are available in a separate publication [26,27].

### Interviews

Students and educators in caring professions who agreed to be interviewed were purposively sampled based on survey results to ensure representation across disciplines, genders, and levels of experience. Semistructured interview guides consisted of open-ended questions designed to explore each participant’s experiences and perspectives on web-based learning aimed at developing virtual caring skills and competencies. Interviews were conducted on the web via Zoom (Zoom Video Communications, Inc) and typically lasted between 20 and 45 minutes. All interviews were digitally recorded and transcribed verbatim for subsequent analysis. Data collection continued until all willing participants had completed their interviews. A total of 9 students and 8 educators participated in the semistructured interviews, which were thematically analyzed using an inductive approach to identify common interactive themes across the data [28,29]. The detailed findings from these interviews are published elsewhere [24].

### Mixed Methods Analysis

Expanding upon our previous efforts and advancing from our prior research, we embarked on a process of cross-referencing our findings across different stages of the study using a mixed methods metamatrix [30]. This integration technique allowed us to comprehensively analyze all gathered data collectively. The implementation of a metamatrix enabled us to develop a robust visual framework, aiding in identifying patterns across various data types and establishing a clear documentation trail [30].

### Consultations With Knowledge Users

Consultations with students and educators occurred iteratively throughout data collection, analysis, and framework development stages. During these consultations, which included presentations at national and international teaching and learning conferences, knowledge users were presented with emerging themes to solicit feedback on the relevance, gaps, and applicability of the framework. The feedback from knowledge users was collated by graduate student team members, shared with the broader research team, and integrated into the final framework.

### Ethical Considerations

The institutional review board that granted the ethics approval for this study is the University of Calgary Conjoing Health Research Ethics Board (REB22-0748). Prior to participation, all participants were provided with a comprehensive consent form that provided a detailed explanation of the study, outlined the voluntary nature of the study, and reinforced participants’ right to withdraw at any time, as well as any associated risks and benefits. All participants were assured that their confidentiality and privacy would be upheld in accordance with the ethics application. Informed consent was obtained from all study participants and no compensation was provided for participation.

## Results

### Overview

**Figure 1.**
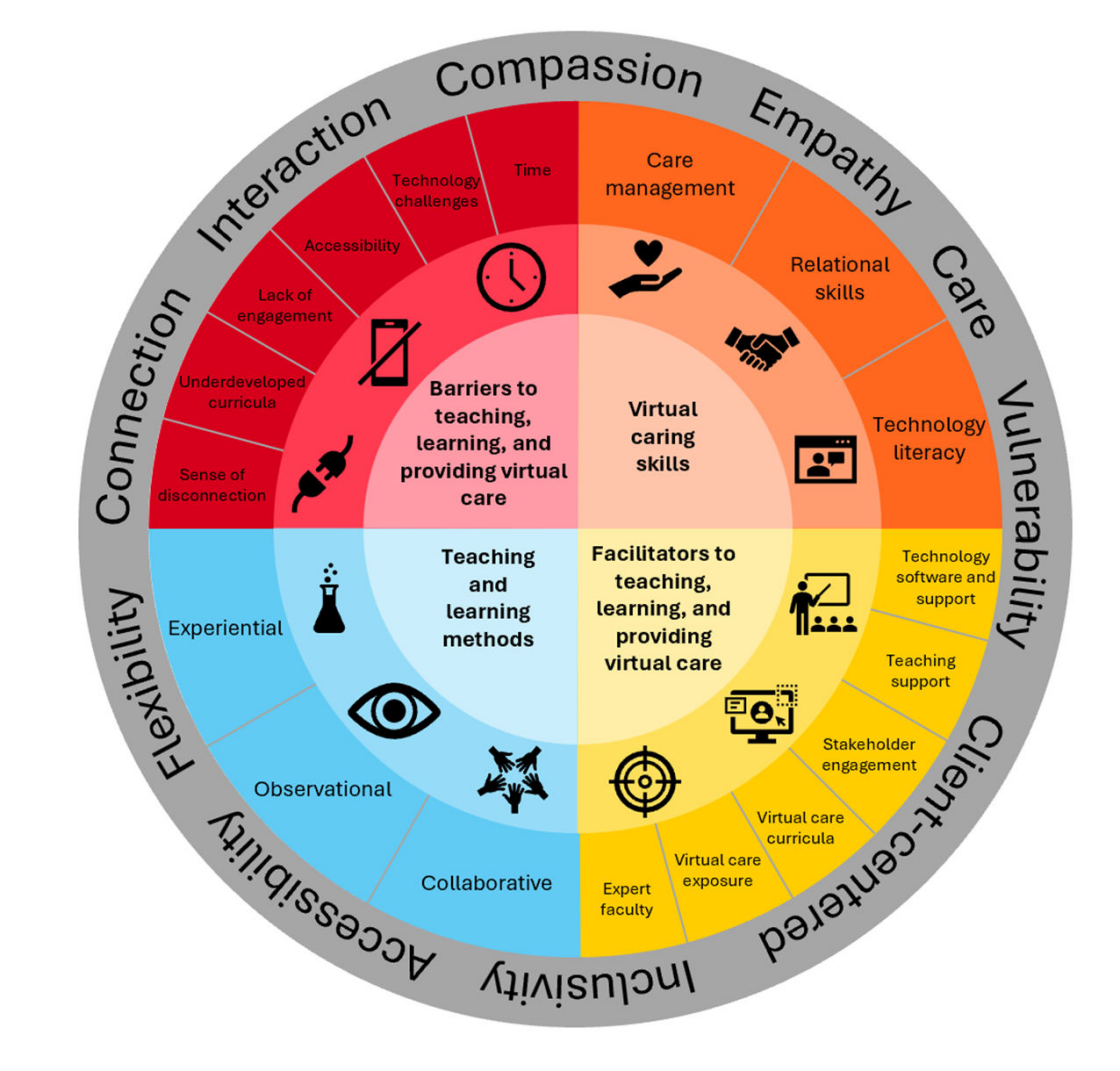
Virtual Caring framework.

The framework developed through this research process (Figure 1) is structured around 10 principles (outer ring) and consists of 4 major domains (center of framework), which are further divided into 18 subdomains. These subdomains encompass key considerations when teaching and learning virtual caring skills in web-based environments.

### Principles for Teaching and Learning Virtual Caring Skills

#### Connection and Interaction

Establishing connections and fostering interactions in virtual environments are crucial for building relationships, as the absence of such connections can noticeably diminish engagement. In virtual settings, dedicating time to understanding the perspectives and concerns of individual students, educators, colleagues, and clients becomes imperative, highlighting the crucial skill of fostering connections for caring professionals. Particularly in the context of virtual learning and caregiving practices, students and educators emphasize the importance of prioritizing personal connections with clients over technical and hands-on skills when delivering virtual care. Various methods, including in-class activities, group work, breakout rooms, allowing time for questions, sharing experiences, role-playing, and using multimodal content, can enhance engagement and interaction. Educators can exemplify effective interaction strategies applicable to providing virtual care.

#### Compassion, Empathy, and Care

Creating learning experiences that prioritize compassion, empathy, and care, particularly in virtual settings, demands emotional labor and deliberate planning. Effectively integrating web-based teaching and learning technologies requires emotional bravery from educators to foster students’ proficiency in virtual caring skills. Designing learning activities that center on social and emotional learning becomes crucial for promoting individual well-being. When it comes to teaching, learning, and offering virtual care, embracing and authentically displaying compassion, empathy, and care are fundamental elements.

#### Vulnerability

In adapting to new virtual environments, both students and educators should be open to exploring unfamiliar approaches, acknowledging that outcomes may not unfold as anticipated. The progress of virtual care may be hindered through a lack of willingness to embrace vulnerability or adopt novel strategies to provide care. Establishing safe and courageous learning and caring spaces is essential. Professionals in the caring field must cultivate a supportive atmosphere within virtual environments, breaking down barriers and nurturing relationships to advance virtual caring initiatives that meet clients’ needs.

#### A Client-Centered Focus

Recognizing the importance of client-centeredness is crucial in virtual caring practices. In web-based learning and interactions, students and clients must perceive a sense of genuine care that prioritizes their well-being. A client-centered approach involves assessing individual client needs and adapting strategies to effectively address these within the virtual environment. This perspective not only informs the questions caring professionals may ask in these situations but also shapes the care provided to future remote clients. The pandemic has heightened our collective awareness of the intricate nature of human experiences, emphasizing the value of adopting a more holistic approach to the teaching and practice of virtual care.

#### Inclusivity and Accessibility

The advent of educational technology has enhanced inclusivity and accessibility in learning and caring, introducing innovative modes of engagement that are particularly beneficial for individuals who are members of marginalized groups. The transformative influence of online and virtual platforms in educational technology has significantly broadened the scope of inclusivity, accessibility, and equity in learning and caregiving. This technological advancement has expanded educational and caregiving opportunities to people residing outside urban areas, or managing diverse life responsibilities, or experiencing physical challenges, providing access that was previously limited. However, accessibility can still be hindered by challenges such as poor internet connectivity, limited web-based or computer literacy or access to appropriate electronic devices, and a lack of physical spaces conducive to web-based learning or accessing web-based care. As virtual caring continues to gain prominence in society and increases accessibility for some, it is important to recognize and support those who may find this mode of caregiving inaccessible.

#### Flexibility

Flexibility emerges as a fundamental principle in acquiring and applying virtual caring skills. The inherent nature of web-based learning provides crucial flexibility, allowing individuals to grasp content and skills at their preferred pace and schedule. This proficiency seamlessly extends to their virtual caring practice, empowering both students and clients to engage more effectively and customize their experiences according to their distinct needs and interests. Demonstrations of flexibility can manifest in various aspects, including accessibility, choice of topics, meeting times, and personalization of care.

### Four Major Domains

#### Virtual Caring Skills

##### Care Management

Effectively managing virtual care requires the cultivation of critical thinking, organizational prowess, advocacy, adaptability, client education, assessment, documentation, and problem-solving skills—all crucial proficiencies for students pursuing careers in caring professions. Profound skills are required to organize client care effectively, conduct thorough client assessments, and advocate for accessing resources within virtual care environments. Caring professionals must excel in asking pertinent questions, discerning when to delve deeper, and adapting skillfully when necessary. These competencies enable them to navigate the complexities of virtual care delivery and ensure comprehensive and compassionate support for clients.

##### Relational Skills

In virtual care settings, fundamental relational skills—including effective communication, relationship building, ensuring psychological safety, and conveying empathy—play a crucial role in providing care. Emphasis must be placed on observing nonverbal cues and voice tone and practicing active listening when operating in virtual contexts. Although establishing rapport and building relationships with clients may present challenges in virtual environments, it remains an essential aspect of care. Nurturing psychological safety and expressing empathy during virtual care delivery can contribute significantly to clients feeling comfortable and at ease throughout the care process.

##### Technology Literacy

Proficiency in technology is vital for delivering virtual care. Students must acquire the skills and confidence to navigate diverse virtual care technology platforms. Being able to operate smoothly within these technological platforms enables students and professionals to redirect their focus to the client and their well-being. Additionally, a comprehensive understanding of the legal and ethical dimensions associated with providing virtual care, including aspects such as thorough documentation and privacy concerns, is of utmost importance.

### Teaching and Learning Methods

#### Collaborative Learning

Collaborative web-based learning plays a pivotal role in fostering the development of virtual caring skills among students in caring professions. Through dynamic mechanisms such as class discussions, group work, and interdisciplinary collaboration, students engage in a rich educational experience that extends beyond individual perspectives. Collaborative learning environments provide a platform for students to share diverse insights, cultivate empathy, and refine their communication skills. In the context of caring professions, this collaborative approach can enable students to understand various dimensions of care, incorporating holistic perspectives from multiple disciplines. Moreover, the virtual nature of these interactions not only mirrors the evolving landscape of care provision but also equips students with digital communication and teamwork skills crucial for contemporary professional practice.

#### Experiential Learning

Experiential web-based learning opportunities offer another avenue for nurturing virtual caring skills among students in caring professions. Engaging in virtual simulations, role-play, and case study scenarios allows students to immerse themselves in realistic yet controlled environments that mirror the challenges and dynamics of their future professions. This hands-on approach not only deepens their understanding of theoretical concepts but also hones their ability to navigate complex situations with empathy and sensitivity. Additionally, virtual practicum experiences can further provide students with authentic interactions with virtual clients. These immersive encounters enable students to apply their knowledge and skills in a safe and supervised web-based setting, fostering the development of virtual caring skills within the context of genuine client interactions.

#### Observational Learning

Observational learning in web-based settings plays a pivotal role in shaping the development of virtual caring skills among students in caring professions. By actively observing faculty members and engaging in virtual settings that simulate professional work, students gain valuable insights into the nuances of compassionate and effective care delivery. This learning approach allows them to witness real-world scenarios, model empathetic behavior, and internalize best practices in virtual care. Through this observational process, students not only grasp the theoretical underpinnings of their field but also cultivate a deeper understanding of the practical application of virtual caring skills. By emulating the actions and techniques demonstrated by experienced professionals in the web-based environment, students can refine their own approach to virtual care, laying the foundation for a compassionate and ethically grounded professional practice in the digital realm.

### Barriers to Teaching, Learning, and Providing Virtual Care

#### Technological Challenges

Certain technological challenges pose significant obstacles to teaching, learning, and delivering virtual care. These challenges encompass issues such as streaming disruptions, navigating unfamiliar technologies, and the inherent unreliability and unpredictability of technology. Caring professionals must acquire the skills not only to use technology themselves but also to guide clients in navigating unfamiliar technological terrain. Moreover, they need to adapt to and address technology failures as they arise. The hurdles associated with technology can have repercussions on both student learning and their capacity to deliver exceptional, appropriate, and timely care to clients.

#### Limited Access to Virtual Caring Equipment, Internet, and Technology

The teaching, learning, and delivery of virtual care can be impeded by challenges such as limited accessibility, inadequate equipment, client web-based literacy, and insufficient internet connections. Scarce access to mobile devices and reliable internet connections not only affects student learning but also restricts the scope of care they can extend to clients, leading to diminished interactivity. These concerns are particularly significant when teaching and delivering virtual care to clients in rural and remote areas, underscoring issues related to unequal access to web-based resources and broader accessibility challenges for clients.

#### Sense of Disconnection

Delivering care in virtual settings may limit the capacity to form personal connections with clients or offer specific therapeutic services that rely on in-person interactions. For instance, therapeutic touch is a caring skill that is typically used in face-to-face interactions. The absence of a personal connection and the difficulty in fostering a sense of community with clients can pose a substantial obstacle to effective care provision. Moreover, delivering appropriately personalized care over the phone presents additional challenges due to the inability to convey messages through body language.

#### Time Constraints

The constraints of time pose significant challenges in the domains of teaching, learning, and delivering virtual care. Both students and clients may struggle to maintain prolonged engagement on the web, while educators face additional demands, especially when facilitating experiential learning for virtual care practices. These constraints underscore the need for efficient and focused approaches to ensure meaningful educational and caring interactions within the limitations of web-based environments.

#### Underdeveloped Virtual Caring Curricular Content

There is a noticeable gap in the availability of content for educators seeking to teach students about virtual caring. Faculty require more resources to adequately prepare students for engaging in virtual caring practices. Often, curricula are constrained and overloaded, with virtual care content treated as an afterthought. Virtual caring skills and competencies, considered specialized practices, may not be conventionally integrated into broader, generalist-focused curricula. However, with the advent of COVID-19 pandemic, virtual care has emerged as an essential skill set for many caring professions. The absence of dedicated curricula on virtual caring could potentially hinder students in caring professions from effectively providing virtual care in their future practices.

#### Lack of Engagement

Interruptions, decisions regarding the use of cameras, and difficulties in connecting with students, educators, colleagues, and clients can diminish levels of engagement. Active participation from clients in education and care settings is required to achieve intended outcomes. In virtual settings, the prevalence of interruptions and uncertainties about whether cameras should be on or off may prompt questions about presence and limit clients’ and caregivers’ overall sense of engagement. Negotiating these challenges is essential for fostering a more conducive web-based environment for providing virtual care.

### Facilitators of Teaching, Learning, and Providing Virtual Care

#### Technology Software and Support

Acquiring knowledge and purposefully using technology and virtual caring software can enhance the facilitation of teaching, learning, and delivering virtual care. Conducting orientation and skill development sessions to familiarize individuals with the technology can be beneficial. Despite the potential assistance of virtual care technology, without a thorough comprehension of its efficient and effective utilization, students and educators may encounter difficulties in establishing meaningful caring connections with clients.

#### Teaching Support

Support for teaching in web-based settings—from teaching and learning departments, colleagues, faculty members, or external networks—can significantly contribute to skill development in virtual care instruction. Continuous development and exchange of best practices serve as valuable avenues for fostering ongoing growth in confidence and competence when using virtual caring technology. Leveraging a collaborative approach ensures a sustained and progressive enhancement of teaching skills related to virtual care in web-based environments.

#### Stakeholder Engagement

Effective development of virtual caring skills relies on support and engagement from various stakeholders, including care providers, clients, students, educators, and institutions. The diversity of perspectives these stakeholders bring to virtual caring experiences is invaluable, and leveraging these viewpoints can enhance the effectiveness of teaching and learning about virtual care. Collaborating with and incorporating insights from these diverse stakeholders are essential for a comprehensive approach to virtual caring skill development.

#### Expert Faculty

Facilitating the learning of virtual caring skills necessitates the presence of expert faculty proficient in web-based education and experienced in delivering virtual care. These experts play a crucial role in modeling and promoting web-based etiquette, fostering psychological safety within the virtual classroom, and structuring interactive activities that contribute to a shared and enriching learning experience.

#### Virtual Care Curriculum

Recognizing virtual care as a current reality underscores the importance of integrating it into professional educational programs focused on teaching caring skills. Enhancing the effectiveness of teaching, learning, and delivering virtual care involves aligning virtual caring expectations, competencies, and learning objectives within the curriculum. Strategies can include developing a dedicated virtual care course or incorporating threaded virtual care topics throughout a curriculum.

#### Virtual Care Exposure

Gaining familiarity with virtual care through activities such as shadowing virtual care providers, practicing virtual caring skills with clients, watching web-based demonstrations, or engaging in role-play and simulated virtual care environments can significantly contribute to developing virtual care proficiency and deepening one’s comprehension of the diverse scope of this mode of practice. Increased exposure to virtual care experiences can enhance the effectiveness of web-based learning.

## Discussion

### Principal Findings

The findings from this multiphase and multifaceted study resulted in the development of an evidence-informed framework to support teaching and learning virtual caring skills and competencies. The components of this Virtual Caring framework are based on our previous research including an evidence synthesis, surveys, and interviews, and the framework was refined through stakeholder consultations. The Virtual Caring framework highlights key principles for virtual care, virtual caring skills, teaching and learning methods, and barriers to and facilitators for teaching, learning, and providing virtual care.

Teaching, learning, and providing virtual care can benefit greatly from incorporating key principles such as connection, interaction, compassion, empathy, care, vulnerability, client-centered approaches, inclusivity, accessibility, and flexibility. Creating a caring environment and cultivating relationships with clients in health care can facilitate shared decision-making, thereby improving client outcomes [31]. In a qualitative study exploring health care providers’ experiences in building rapport in telehealth settings, caring professionals emphasized that building rapport with clients is essential for fostering positive, productive interactions with clients and their families [32]. Additionally, establishing meaningful connections and interactions in virtual environments is essential for nurturing a sense of community and trust among students [33]. Felton et al [33] stated that trust is fundamental in the educator-student relationship and crucial for students to feel psychologically safe. They recommended *trust moves* to help foster the trust of students, including strategies such as getting to know the students, demonstrating mastery of the content, engaging respectfully, and showing sensitivity to diverse identities [33].

Infusing compassion, empathy, and care into virtual interactions ensures that the human element remains integral in digital spaces, promoting a more client-centric and supportive care environment. Recognizing vulnerability as a strength rather than a weakness encourages open communication and collaboration, thereby deepening connections between care professionals and clients. Adopting a client-centered approach ensures that virtual care is tailored to each individual’s unique needs and preferences, promoting the effectiveness and personalization of interventions. In a scoping review examining barriers and facilitators to telehealth implementation in populations at risk for health disparities, Baily et al [34] indicated that client-centered approaches serve as facilitators in delivering telehealth care and can help mitigate health disparities. Moreover, Health Canada [35] has underscored the importance of a client-centered and community-centered focus to achieve the quadruple aim in telehealth. Inclusivity and accessibility principles can ensure that virtual care remains accessible to everyone, regardless of diverse backgrounds, life situations, or abilities. Finally, flexibility enables adaptation to the evolving landscape of virtual care, accommodating the dynamic needs of both learners and clients. By embracing these principles, educators, learners, and care providers can create virtual care environments that are not only effective and efficient but also empathetic, client-centered, and inclusive.

To effectively provide virtual care, students in caring professions require specific skills. Robust care management skills are paramount to ensuring that caring professionals can organize, coordinate, and optimize the delivery of care in the digital landscape. This includes proficiency in using virtual platforms for scheduling, monitoring client progress, and facilitating communication among the care team. Relational skills are equally important, enabling care providers to establish and maintain meaningful connections with clients through virtual channels. This involves cultivating effective communication, active listening, and empathy to create a supportive and trusting virtual care environment. Noddings [36,37], an education scholar, discussed the centrality of relationships and caring in education, emphasizing the importance of listening carefully and responding to expressed needs without assuming what students may require. The theoretical writings of Noddings [36,37] are applicable across various caring professions and can guide professionals in delivering client-centered care that is supported by relational skills. Additionally, technology literacy is foundational in virtual caring, equipping care providers with the competence to navigate and leverage digital tools effectively. Combining these skills enables the delivery of high-quality virtual care that is not only technologically proficient but also rooted in the principles of empathy, communication, and client-centeredness.

Collaborative, experiential, and observational learning form a robust triad that supports students in developing essential virtual caring skills. Through collaborative learning, students engage in collective exploration and discussion, fostering a diverse range of perspectives and insights that contribute to a holistic understanding of virtual care. Experiential learning, which includes virtual simulations and practical scenarios, allows students to actively apply theoretical knowledge in realistic contexts, enhancing their problem-solving abilities, and deepening their virtual caring skills. In a study examining the impact of virtual telehealth simulation on prelicensure community health nursing students, significant increases in knowledge, attitude, and confidence were observed following simulation-based education [38]. Students also rated the learning experience highly and found the content important to their learning [38]. Although virtual simulations have gained popularity in health care, this experiential learning modality may also prove valuable in other caring professions, such as social work [39]. Observational learning can further complement these approaches by providing students with the opportunity to witness seasoned professionals in action. This allows them to model empathetic behavior, effective communication, and other crucial skills in virtual care settings. Moreover, students need to see their educators as caring professionals who provide an environment conducive to their learning. This combined approach not only enhances students’ proficiency in using digital platforms but also cultivates the interpersonal and technical competencies required for successful and compassionate virtual care practice.

Several challenges can pose significant barriers to the effective teaching, learning, and provision of virtual care. Technological challenges can impede the seamless delivery of virtual care services and teaching and learning experiences. According to Ortega et al [40], limited access to the internet or technological devices can exacerbate disparities, preventing some individuals from participating in web-based learning or accessing virtual care. This limitation can create a pervasive sense of disconnection, stemming from the absence of face-to-face interactions, which affects both student engagement in learning and clients’ perception of care. Time constraints further compound these issues, as professionals and learners may struggle to balance virtual care demands with other responsibilities. Moreover, maintaining engagement poses a challenge, as the virtual environment may not always capture the same level of attention and participation as in-person settings. Additionally, the underdeveloped nature of virtual caring curricula poses a barrier. Educators grapple with the task of aligning educational materials with the dynamic and evolving landscape of virtual care, potentially leaving gaps in essential skills and knowledge. Addressing these challenges is crucial for ensuring that virtual care education is inclusive, effective, and capable of meeting the diverse needs of both learners and care recipients.

The effective facilitation of teaching, learning, and the provision of virtual care hinges on several key factors. First and foremost, technology software and robust technical support are crucial in creating seamless virtual care experiences. Access to reliable platforms and assistance in navigating digital tools ensure that caring professionals and students can confidently engage in virtual care settings. Teaching support also plays a critical role. Educators benefit from training in web-based pedagogy and virtual care methodologies, which enhances the quality of instruction and fosters an environment conducive to optimal learning outcomes. Moreover, active stakeholder engagement is essential. Collaboration with care organizations, policy makers, and industry experts helps align virtual care education with real-world practices and expectations. Expert faculty with specialized knowledge in virtual care enriches the learning experience by offering valuable insights and mentorship. Exposure to diverse virtual care scenarios through simulation or practicum further enhances preparedness. Learners can apply theoretical concepts in practical settings and adapt to the complexities of digital care delivery [41]. In concert, these elements create a supportive framework that empowers both educators and learners to navigate the nuances of virtual care with confidence and proficiency.

### Practice Implications

As workforce needs continue to shift, our Virtual Caring framework can support the development of virtual caring skills and competencies across various caring professions. We created this framework to serve as a tool for educators, students, and decision makers, enabling the systematic and intentional integration of virtual caring skill development into caring professions education. We anticipate that our framework will be useful to several target audiences across multiple organizational levels, including educators, students, decision makers, and practice partners. For example, educators and students can use this framework to facilitate discussions on teaching and learning strategies, establish plans for enhancing virtual caring skills, and promote critical reflection and career growth in virtual care practices. Institutional decision makers and teaching and learning centers can leverage the framework to guide the development of programs tailored to nurturing virtual caring skills among professional students. At a broader level, institutional, regional, national, and international associations can use the framework to amplify conversations and develop strategies aimed at fostering widespread cultural shifts in integrating virtual caring skills into caring professions education. Despite potential resource constraints in higher education institutions related to virtual care technology and teaching support, our framework offers a structured approach for administrators to strategically plan virtual caring initiatives that address the growing demands of caring professions. Additionally, educators can identify opportunities for collaboration with other caring professional programs, potentially creating synergies that enhance program offerings. Thus, our Virtual Caring framework catalyzes advancing the integration of virtual caring competencies across caring professions education, ensuring preparedness and responsiveness to evolving workforce needs.

### Strengths and Limitations

This research reflects the global state of evidence on web-based learning opportunities designed to develop and apply virtual caring skills and competencies among students in caring professions. Our research, while underpinned by global literature, primarily focused on the perceptions and experiences of caring professions students and educators from one Canadian university, aiming to provide insights across various disciplines. The inclusion of participants from a range of disciplines strengthened this study, allowing for a deeper exploration of the complexities involved in web-based learning for developing virtual caring skills. The triangulation of data from different phases of this study into an evidence-informed framework strengthens the findings.

While our research followed a systematic and rigorous approach, this study is not without limitations and caveats. Despite a comprehensive and systematic literature search, some relevant literature might have been missed. Additionally, variations in web-based teaching and learning methods regarding virtual caring skills posed challenges for direct comparisons, yet they also provided valuable insights into different approaches, benefits, and associated challenges. Moreover, although the literature informing this work is globally representative, the data collection from student and educator surveys and interviews was conducted at one research-intensive university in Canada, potentially limiting the generalizability of our findings to other institutions. However, stakeholder consultations at 3 different national and international events provided a broader perspective and validated the relevance of the framework beyond the study’s specific context.

### Future Research

Our study also underscores the need for further research with robust study designs to better understand how to effectively support the development and implementation of web-based learning opportunities that help students in caring professions in developing the required virtual caring skills and competencies. Existing research on teaching virtual caring skills in web-based settings may not be robust [41]; however, the need for caring professionals to develop virtual caring skills and competencies is clear [24,26]. Our comprehensive framework designed to facilitate the development and integration of web-based learning opportunities aimed at nurturing essential virtual caring skills across caring professions is novel. Rigorous evaluation of the use and impact of this framework is needed [41]. Future longitudinal studies could also investigate the sustained impact of virtual caring skills development over time.

### Conclusions

This evidence-informed Virtual Caring framework was developed by educators for educators, specifically to support web-based teaching and learning of virtual caring skills and competencies among caring professional students. It serves as a tool for planning, promoting, and enhancing the development of virtual caring skills in web-based educational settings. Institutions can use this framework to inform their efforts in refining current strategies or developing new approaches aimed at fostering virtual caring skills development among their students.
